# Orthodontic camouflage treatment of a hyperdivergent adolescent patient with anterior open bite and TMD: a case report

**DOI:** 10.1186/s12903-024-04264-z

**Published:** 2024-05-28

**Authors:** Yilin Jiang, Yajing Wang, Tianqi Wang, Dongqiao Liu, Chen Lin, Jing Wang, Cheng Zhi, Ziqian Qiu, Yuanfu Hou, Chunxiang Zhang

**Affiliations:** 1 Department of Orthodontics, School of Medicine, Tianjin Stomatological Hospital, Nankai University, Tianjin, China; 2 Tianjin Key Laboratory of Oral and Maxillofacial Function Reconstruction, Tianjin Stomatological Hospital, Nankai University, No. 75 Dagu North Road, Heping District, Tianjin, 300041 China; 3 School and Hospital of Stomatology, Tianjin Medical University, Tianjin, China; 4 Department of Stomatology, Peking University BinHai Hospital, Tianjin, China; 5 Department of Orthodontics, Zhengzhou Stomatological Hospital, Zhengzhou, China; 6 Department of Stomatology, Shanxi Bethune Hospital, Third Hospital of Shanxi Medical University, Taiyuan, China

**Keywords:** Orthodontic treatment, Anterior open bite, MEAW, Temporomandibular joint, Deleterious oral habits, Occlusal plane

## Abstract

**Background:**

In orthodontics, anterior open bite is a common malocclusion that recurs frequently. Because the causes of anterior open bite are so varied, medical professionals must create customized treatment programs for each patient based on their unique etiology. Through the lowering of the posterior teeth, closure of the anterior teeth gap, and cooperation with intermaxillary traction, the treatment plan outlined in this case study sought to achieve a stable occlusion.

**Case presentation:**

This case report aims to describe an orthodontic camouflage treatment of a 15-year-old female patient with anterior open bite, arch width discrepancy and a history of temporomandibular joint disorder. The patient was treated with intermaxillary vertical elastics and the multiple edgewise arch wire (MEAW) approach. A satisfactory occlusion with a neutral molar relationship was attained after 29 months of orthodontic therapy. The condylography recording showed that this patient’s occlusion tended to be more stable both before and after our treatment. The purpose of this case study is to provide an overview of an orthodontic camouflage treatment for a female patient, who had a history of temporomandibular joint disease, anterior open bite, and arch width disparity.

**Conclusions:**

Our results demonstrated that more attention should be paid to levelling the occlusal plane, intrusion of the molars, decompression of temporomandibular joints and the etiology factors of malocclusion during the orthodontic period for those patients with anterior open bite.

## Background

The prevalence of skeletal Class II malocclusion in Asian population is approximately 9.91% [1]. Many researchers have found that anterior open bite is common in Class II malocclusion and has many complex etiologies. Anterior open bite is a common malocclusion in orthodontic clinical treatment that has a major impact on oral function, facial aesthetics and temporomandibular joint (TMJ) function. Anterior open bite is characterized by a lack of occlusal contact between the anterior teeth and an irregular deformation in the three-dimensional direction of length, width and height. It has also been identified as one of the most important triggering factors for temporomandibular joint disorders. Idiopathic condylar resorption (ICR) is a progressive resorption of the TMJ heads for unknown reasons, which is common in patients with Class II skeletal hyperdivergent disorder. Clinical manifestations include a decrease in the height of mandibular ramus, clockwise rotation of the mandible, mandibular retrusion and progressive anterior open bite. Adolescent females are more frequently affected by ICR. Special considerations must be made when conducting orthodontic treatment for these patients.

Anterior open bite is a multifactorial condition whose causes are so complicated that they include oral habit components, dental problems and skeletal anomalies [2]. To our knowledge, we need to develop different treatment plans depending on the specific etiology of these patients. In children, early preventive orthodontics, such as blocking bad oral habits, is very important. It is easy to control the growth of the face during childhood. However, since it is not possible to affect the skeletal pattern, the treatment of anterior open bite in adults is very difficult [3]. The golden standard of treatment for anterior open bite is combined orthodontic and orthognathic treatment.

Some researchers have reported that groups with anterior open bite always have an abnormal occlusal plane. Leveling the occlusal plane in patients with anterior open bite is a challenge, especially in controlling the vertical height of the posterior region. Molar intrusion in the treatment of anterior open bite has been a proposed treatment option for many years. Traditional methods such as MEAW, elastic traction and orthodontic extraction treatments are included in the formulation of the corrective treatment plan. The reconstruction of the occlusal plane can be performed with a force system that originates from the tip back of the arch wires. Recently, miniscrews are often used as temporary anchorage in orthodontic treatment [4, 5]. These techniques can provide the anchorage required for tooth movement.

This case report describes the case of a patient with an anterior open bite who underwent non-surgical orthodontic camouflage treatment and the MEAW technique.

## Case presentation

### Diagnosis and etiology

A 15-year-old female patient visited the Department of Orthodontics at Tianjin Stomatological Hospital with the chief complaint of an anterior open bite. In addition, she also had a problem with her bilateral temporomandibular joint. She wanted to correct her occlusion through orthodontic treatment.

**Fig. 1:**
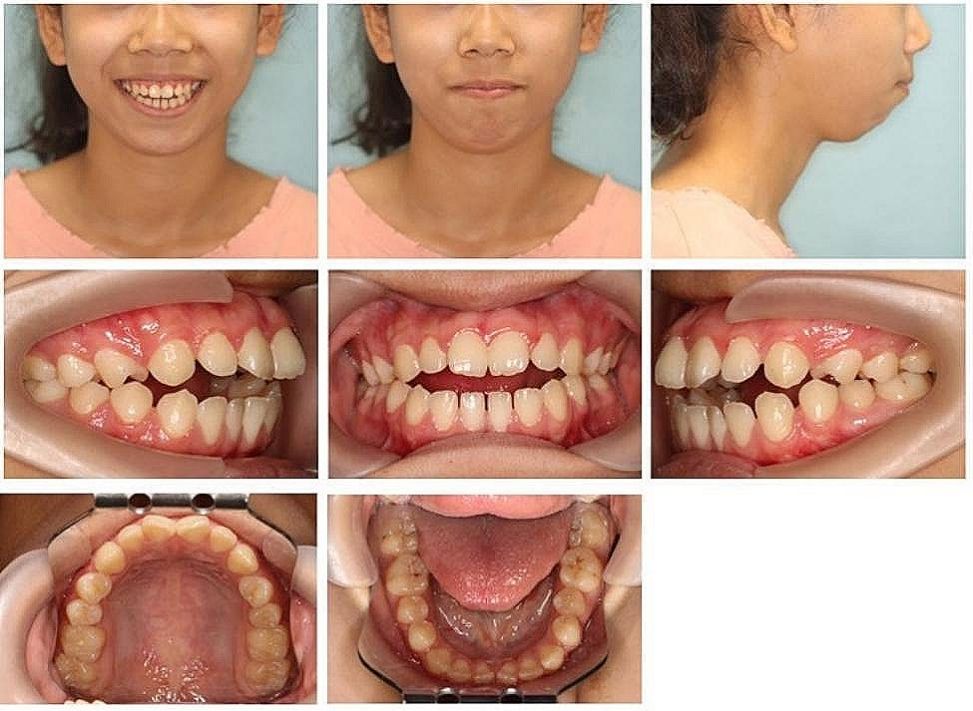
Pretreatment facial and intraoral photographs

**Fig. 2:**
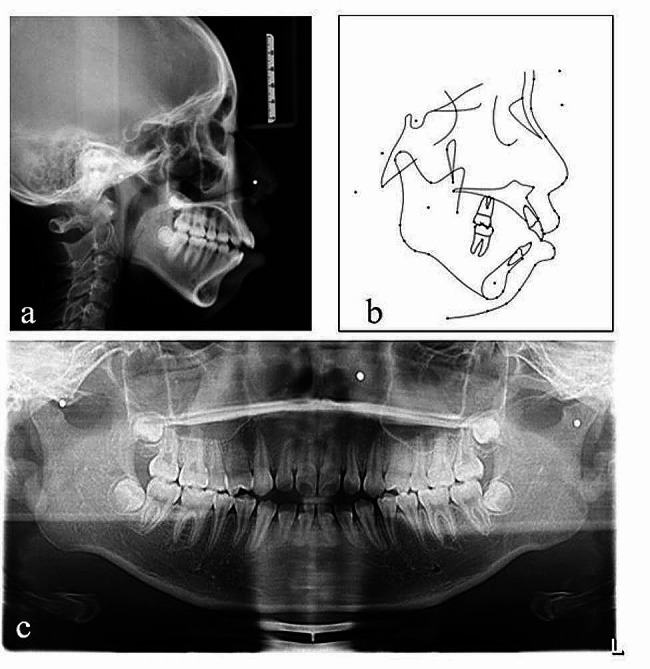
Pretreatment radiographs: **a** lateral cephalogram, **b** Pretreatment lateral cephalogram tracing, **c** panoramic radiograph

The asymmetry of the face with a chin deviating to the right was observed on the frontal facial photograph (Fig. 1). The lateral facial photograph showed that the patient’s jaw was retracted and the mento labial sulcus was slightly flattened. Her maxillary dental midline was in line with the facial midline and the mandibular dental midline was shifted 1.0 mm to the right. Intraoral photographs showed an open bite measuring 3.4 mm. The overjet was 8.4 mm. The patient presented with a Class II molar relationship on the right side, and a Class III molar relationship on the left side. Class II canine relationships on both sides. The dental space discrepancies were 5.5 mm in the maxillary arch and 4.5 mm in the mandibular arch (Fig. 2). According to the cervical vertebral maturation method (CVM), the girl was classified as CVM stage 5, and the peak of growth and development had been completed for at least one year.

**Fig. 3:**
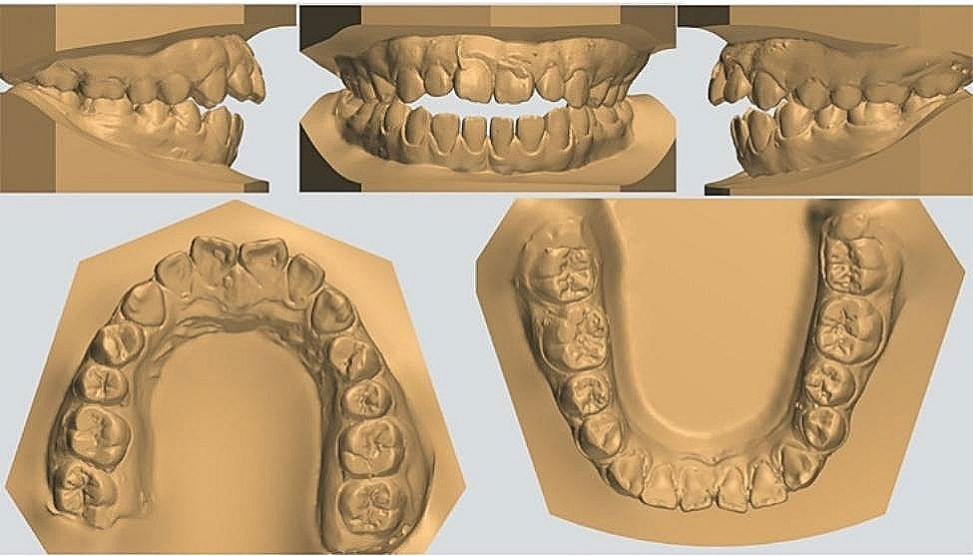
Pretreatment dental casts

The panoramic radiograph showed the presence of all teeth, including the third molars in the budding stage (Fig. 3). Cephalometric analysis (Table 1) showed a skeletal Class II malocclusion (ANB = 10.3°;Wits = 3.1 mm). The vertical facial pattern was hyperdivergent (MP-SN,40.0°, S-Go/N-Me:60.1%). The axial inclination of the mandibular incisors to the mandibular plane exceeded the normal value, indicating that mandibular incisors were inclined labially.

**Fig. 4:**
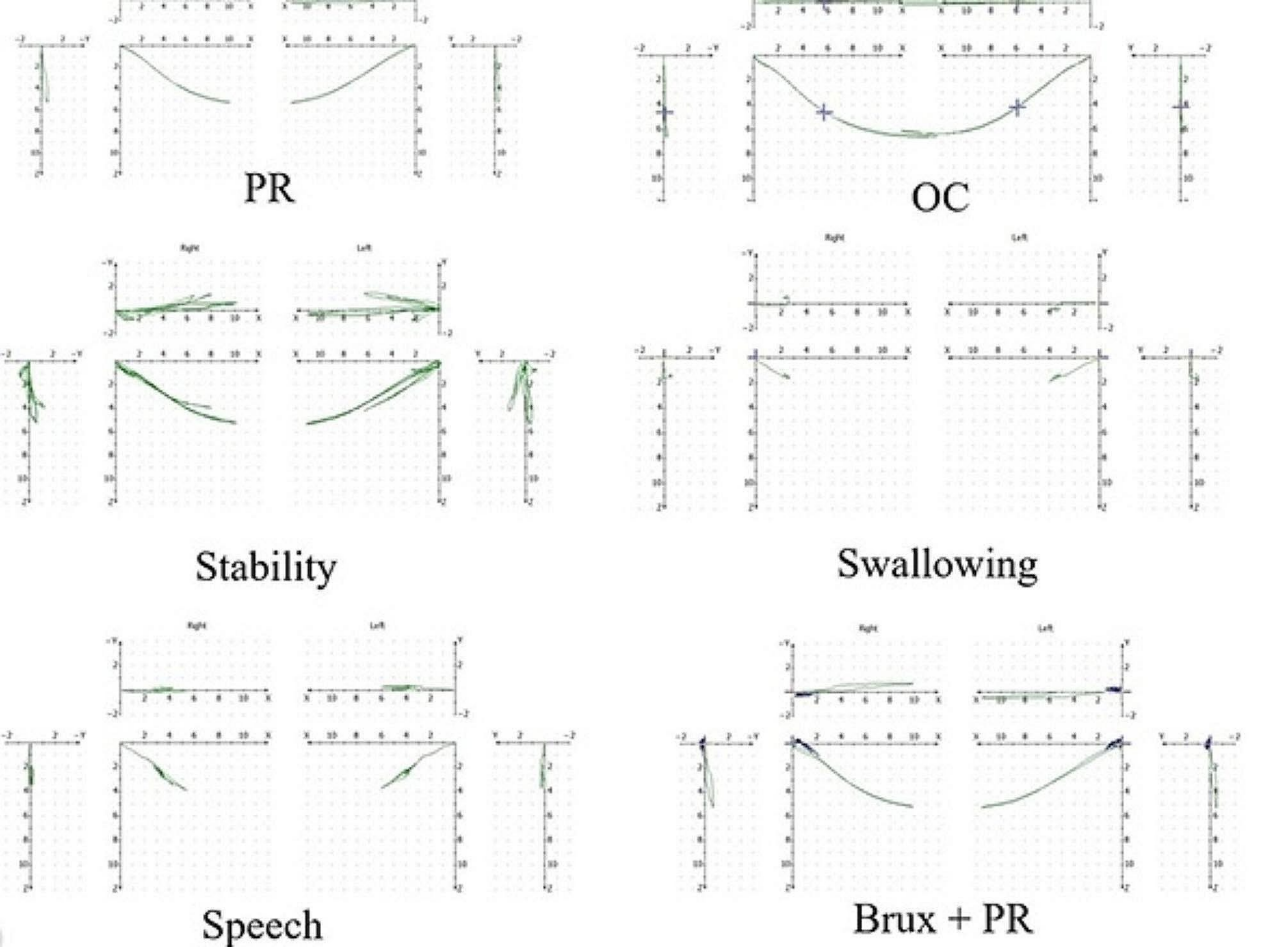
Pretreatment analysis of condylar trajectory

The analysis of the condylar trajectory showed that the bilateral short condylar border movement is within the normal range. However, the condylar trajectory indicated that the functional movement is impaired. At the same time, the stability of the bilateral condyles is poor (Fig. 4).

The patient’s diagnosis was Class II malocclusion with moderate interdental space, moderate protrusion of the maxilla, slight retrognathism of mandibular and severe chin retraction. Genetics and poor oral habits may play an important role in the underlying skeletal pattern and dental anomalies.

### Treatment alternatives

Considering the great improvement in the patient’s profile, growth modification and tooth alignment, we offered this patient two alternatives. The first alternative was a combined orthodontic-orthognathic treatment. As the patient is not yet 18 years old, we advised her to opt for this option when she is an adult in order to achieve the great improvement of her profile and occlusion. The second alternative was an orthodontic camouflage treatment with no extractions except for the third molars. In this option, we pretended to use multiloop edgewise arch wire (MEAW) technique to upright and lower posterior teeth. To improve the open bite of the anterior teeth, reverse-curve arch wire and vertical elastics in the anterior region is necessary. Teeth #38 and #48 should be extracted to make room for tooth movement. If necessary, miniscrews are used for intruding posterior tooth.

In fact, a combined orthodontic-orthognathic treatment was recognized as the best alternative for this patient to improve her esthetics and occlusal function. However, the patient and her family strongly rejected option 1 and preferred the orthodontic camouflage treatment instead. They also accepted the possibility of option 2, that could not achieve the desired profile. Ultimately, the second option was chosen and accepted by the patient after she had fully considered the potential risks and outcomes.

### Treatment progress

**Fig. 5:**
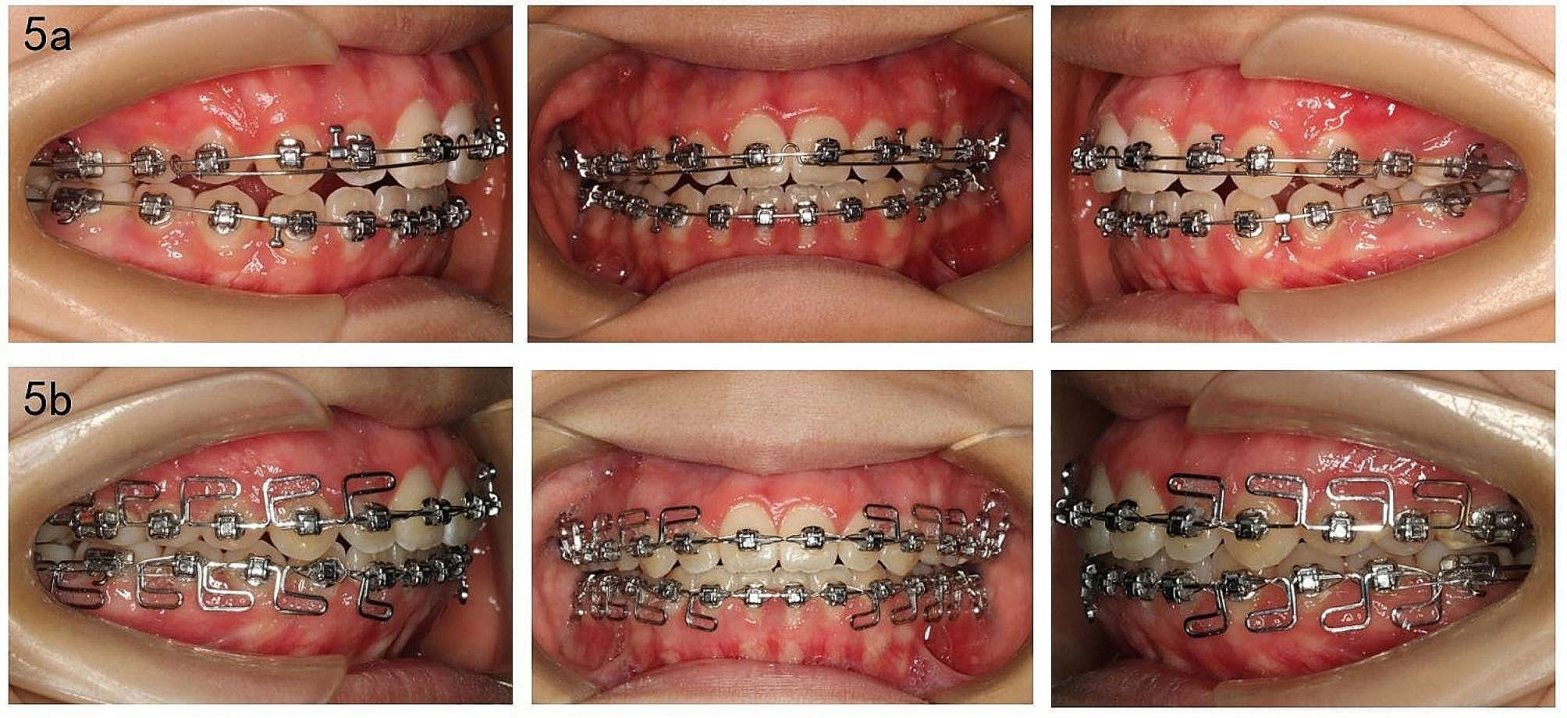
Progress intraoral photographs. **a** The assistant expansion arch was attached to those main arch wires from tooth#13 to tooth #23 for expanding the anterior arch width. **b** Stainless steel arch wires of 0.017 × 0.025-inch were bent into multiloop edgewise arch wire, and we engaged it with slots, meanwhile with up-and-down elastics at the anterior part

A preadjusted fixed appliance with 0.022 × 0.028-inch slot (Damon Q U/L 5–5 Standard Torque, Brea, Calif) was placed in both arches. Initially, the teeth were leveled with a sequence of 0.013-inch and 0.016-inch nickel-titanium wire for 4 months. The patient was unable to seek timely follow-up for the reason of COVID-19 and the aligning stage lasted for up to 5 months. During the leveling phase, 0.014 × 0.025-inch rectangular nickel-titanium wire, 0.016 × 0.025-inch rectangular nickel-titanium wire, and 0.018 × 0.025-inch rectangular nickel-titanium wire were sequentially applied. At this stage, the residual spaces were closed by elastic chain wiring at the phase of 0.018 × 0.025-inches rectangular nickel titanium wire. To expand the width of the anterior maxillary arch, offsets at tooth #13 and tooth #23 were added to the arch wire. After 13 months of treatment, an assistant expansion arch wire was attached to these main arch wires from tooth#13 to tooth #23 to expand the width of the anterior arch (Fig. 5a). The patient was also asked to wear up-and-down elastics for correcting anterior teeth open bite in this phase. Last but not least, myofunctional therapy was recommended to eliminate the tongue-thrusting habit from beginning to end. We instructed the girl to click her tongue against the upper palate and make a sound. When the patient initially instructed the tongue movement, she trained the tongue to lift and make contact with the upper palate. In the intense adjustment of the end stage in the treatment, we applied multiloop edgewise arch wire 0.017 × 0.025 stainless steel in the maxilla and a multiloop edgewise arch wire 0.017 × 0.025 stainless steel were used in the mandible, Up-and-down elastics (1/8-inch 3.5 oz) were applied to retract and extrude the maxillary anterior teeth at this phase (Fig. 5b).

The entire orthodontic treatment process lasted for 29 months. Class I molar and canine relationships with adequate overbite and overjet were achieved. In the meantime, the appliances were removed. The maxillary and mandibular teeth were then stabilized with orthodontic film-pressure transparent retainer.

**Fig. 6:**
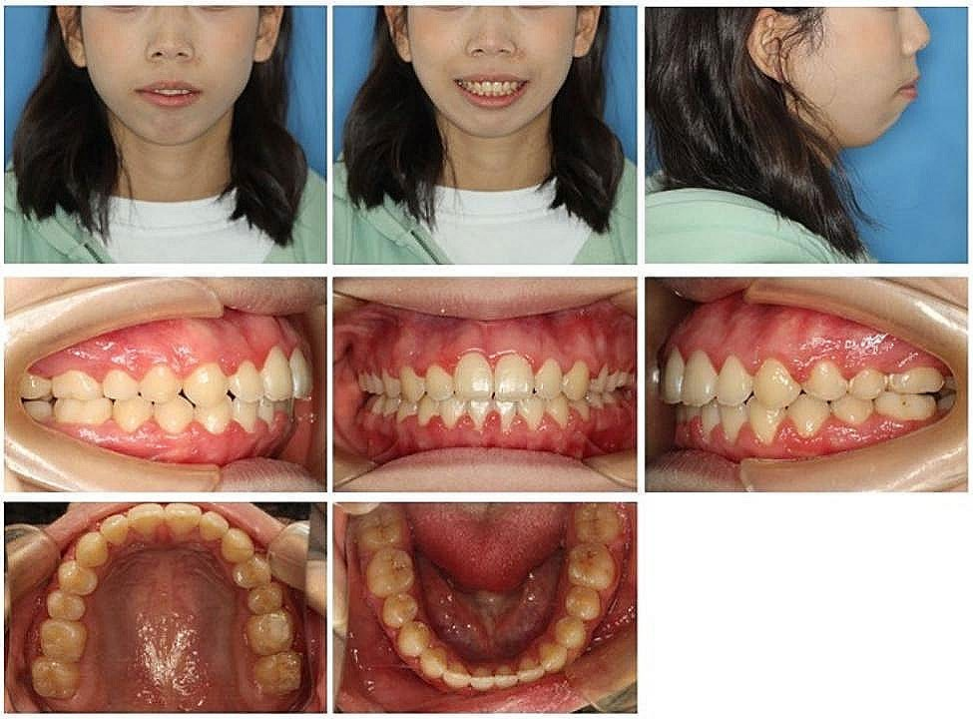
Posttreatment facial and intraoral photographs

**Fig. 7:**
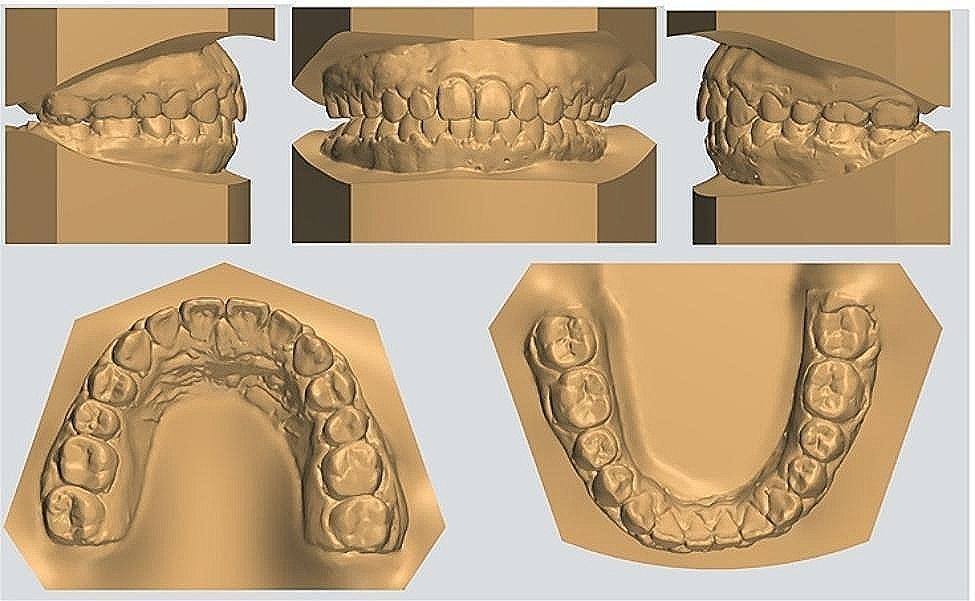
Posttreatment dental casts

**Fig. 8:**
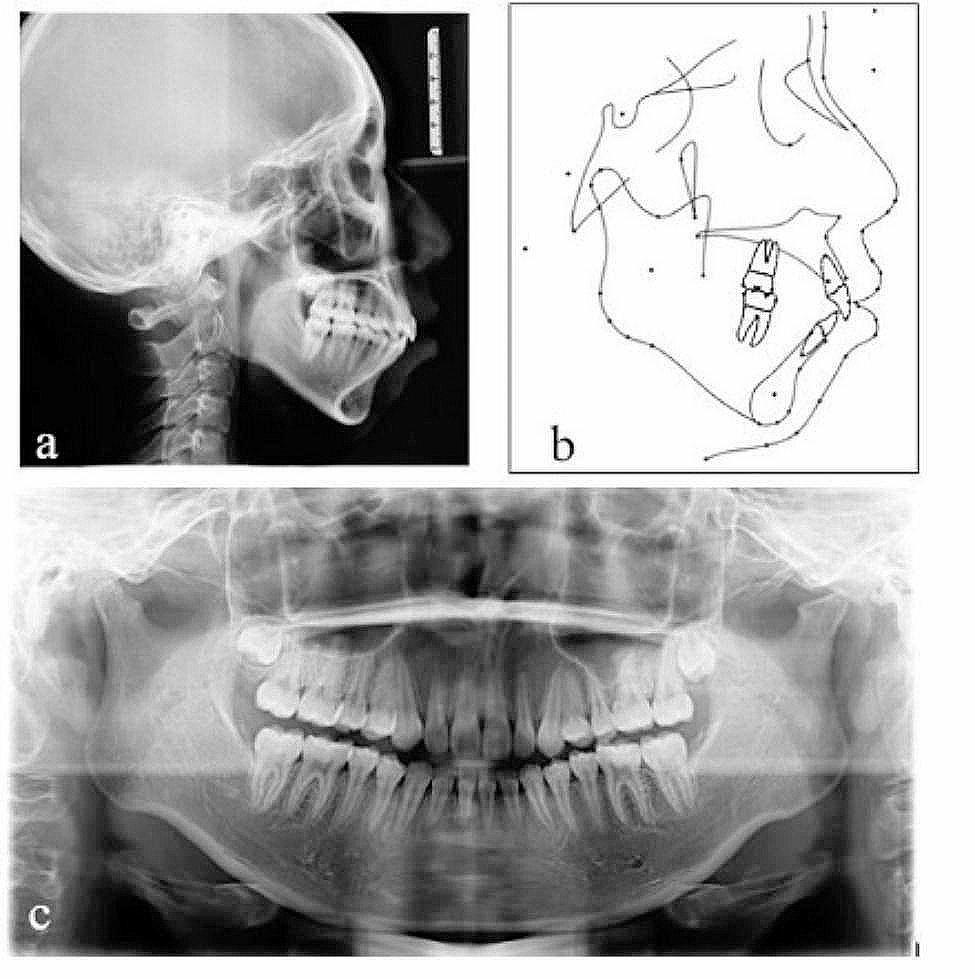
Posttreatment radiographs: **a** lateral cephalogram, **b** Pretreatment lateral cephalogram tracing, **c** panoramic radiograph

**Fig. 9:**
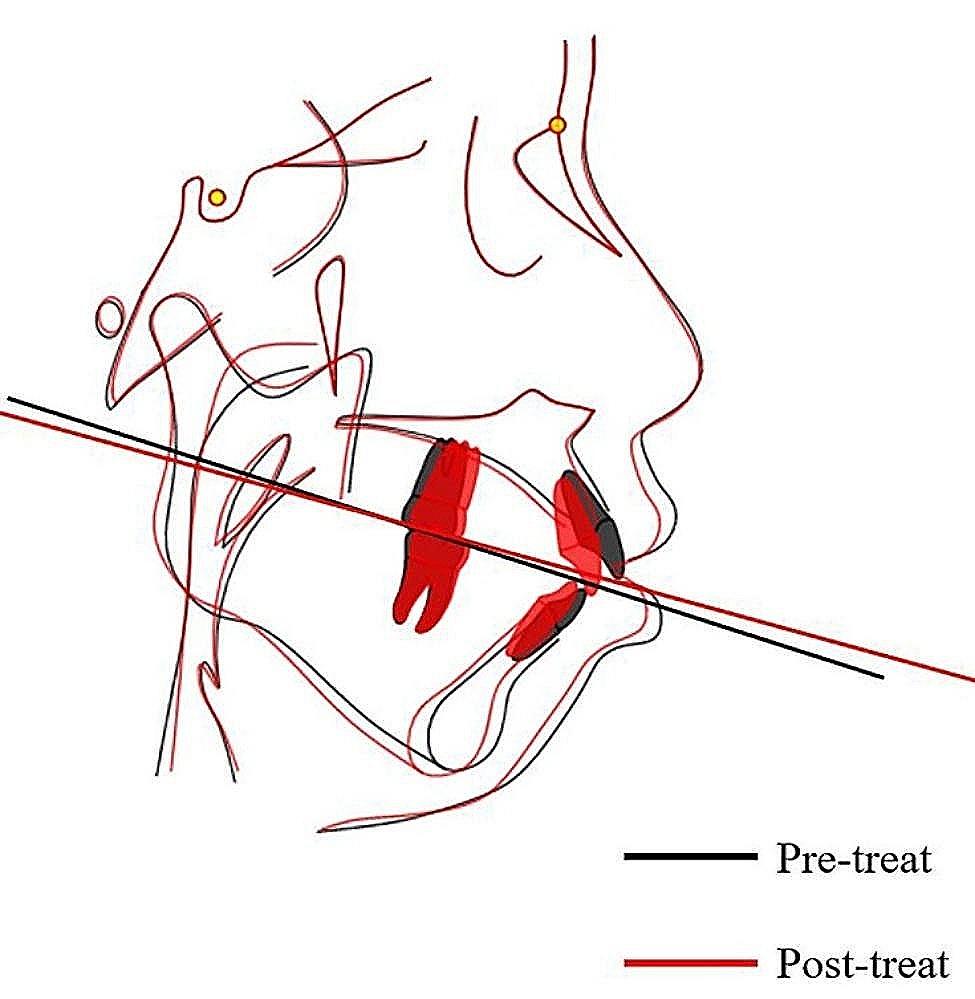
Cephalometric superimpositions. Black line: pretreatment, Red line: posttreatment

After 29 months of orthodontic treatment, Class I molar and canine relationship was achieved. The post-treatment records showed well-aligned teeth in both arches with tight interdigitation (Figs. 6 and 7). Adequate overjet and overbite were achieved. The protrusion was greatly improved to a certain degree and the patient was satisfied with the treatment result. The Post-treatment panoramic radiograph showed satisfactory root parallelism and dental space closure(Fig. 8). The cephalometric analysis showed a slight counterclockwise rotation about occlusal plane (OP-MP, from 21.1° to 20.4°；PP-OP, from 23.5° to 22.4°), a flat occlusal plane was achieved. The ANB improved from 10.3° to 8.4° (Table 1；Fig. 8；Fig. 9).

**Fig. 10:**
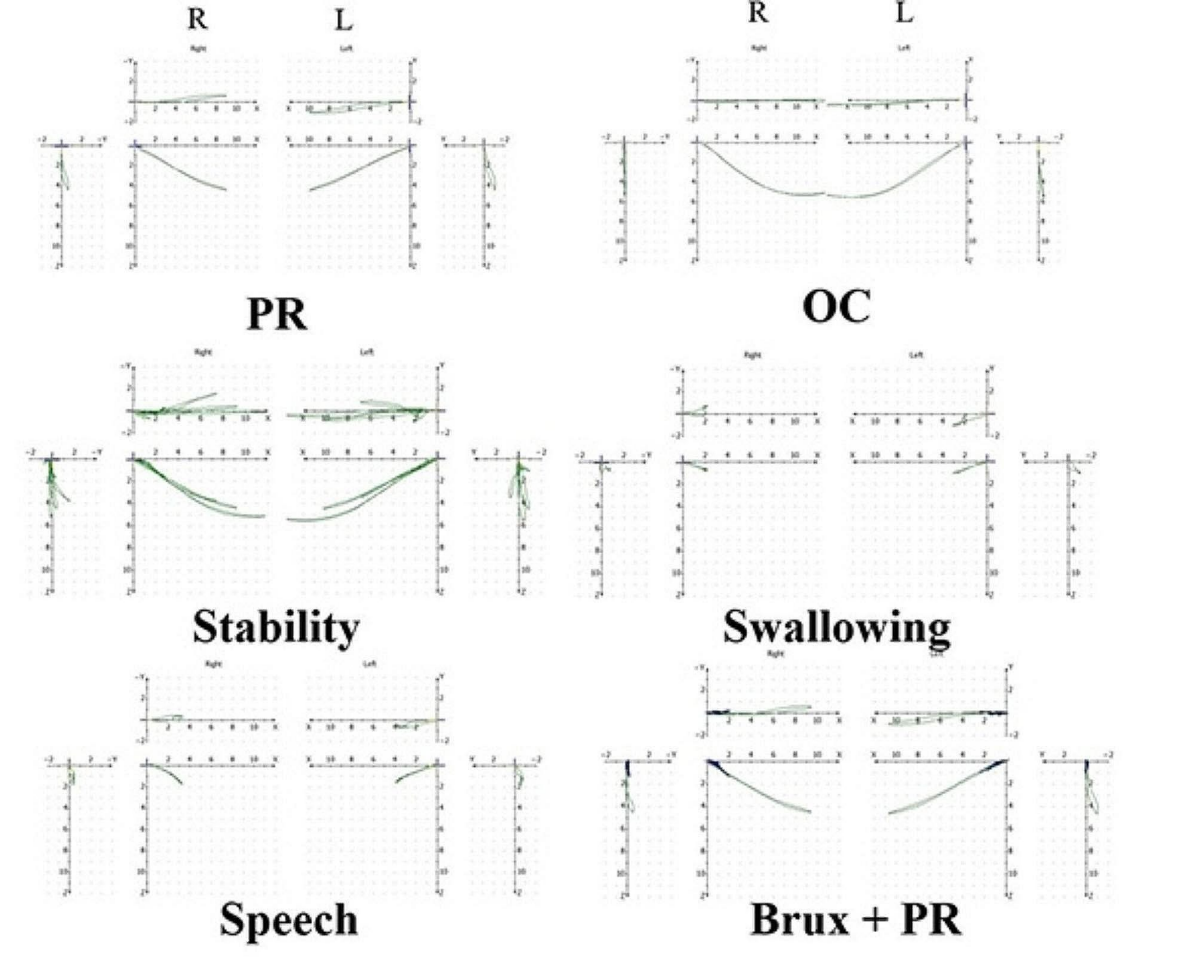
Posttreatment analysis of condylar trajectory

After the appliance had been removed for a month, the patient had no complaints in the TMJ. We performed a condylar trajectory analysis on the patient. The patient showed stable functional movement of the mandible. The result showed that the border movement of the mandibular remained within the normal range (Fig. 10).

**Table 1:** Cephalometric Measurements

Measurements	Normal	Pretreatment	Posttreatment
SNA,°	84 ± 3	87.3	85.6
SNB,°	80 ± 3	77	77.2
ANB,°	4 ± 2	10.3	8.4
SN-MP,°	35 ± 4	40	43.8
U1-L1,°	121 ± 9	108.4	122.1
U1-SN, °	107 ± 6	103.5	92.4
L1-MP, °	95 ± 7	100.4	97.9
U1-NA, °	24 ± 6	21.1	10.5
L1-NB, °	32 ± 6	37.4	39
S-Go/N-Me, %	67 ± 4	60.1	61.1
Y- axis, °	65 ± 4	76.2	78
U1-NA, mm	4 ± 2	5.3	1.4
L1-NB, mm	7 ± 3	10.7	10.5
Wits appraisal, mm	1 ± 1.5	3.1	0.2
PP-OP, °	10 ± 4	23.5	22.4
MP-OP, °	17.4 ± 5	21.1	20.4

## Discussion

Recently, anterior open-bite is one common kind of malocclusion, which has an adverse effect on oral function, temporomandibular joint and facial aesthetics. At the same time, it also has an impact on the physical and mental health of patients. Anterior open bite is a type of malocclusion caused by abnormal vertical relationship between teeth, alveolar bone, or jaw bone. Previous literature has indicated that the etiology of open-bite is complicated. Besides, the treatment of open-bite is of great difficulty, which also has the possibility of recurrence [6].

Before the orthodontic treatment, we conducted a detailed examination of the patient. Many bad oral habits were identified, such as sticking out the tongue, breathing through the mouth and chewing sideways. In the habit of spitting out the tongue, the anterior region is open and the gap is spindle-shaped, which corresponds to the shape of the tongue [7]. Once an open bite occurs, it can also lead to secondary bad tongue habits and a vicious cycle that aggravates the deformity of open bite. Therefore, during the follow-up of orthodontic treatment, we should pay attention to the patient’s chewing movement and check the functional movements of the tip of the tongue, the back of the tongue, and the tongue muscles [2]. The myofunctional therapy in this case report mainly focuses on training tongue muscle function. We instructed the girl to click her tongue against the upper palate and make a sound. When the patient initially instruted the tongue movement, she trained the tongue to lift and make contact with the upper palate. Apart from this, tongue lifting training coordinated with gum chewing may have a greater effect. Tongue lifting training has the function of correcting the shape of the maxillary arch and alveolar arch, which can maintain the long-term stability of the corrected arch shape.

The Spee curve is one of Andrews’ six elements of orofacial harmony that should be corrected to the normal range during orthodontic treatment [8]. Andrew also stated that leveling and flattening of the Spee curve should be the goal of treatment. A normal Spee curve is not only an important factor in achieving stable and close occlusion, but also ensures that the dentition can withstand strong occlusal force during mastication [9]. The fulcrum effect formed by premature contact changes the position of the condyle within the joint fossa, ultimately leading to TMD [10]. Therefore, orthodontic treatment should restore the appropriate curvature of the Spee curve, ensure proper tooth inclination and good intercuspal occlusion. The mandible can achieve stable forward movement guided by the anterior teeth, and the posterior teeth can separate quickly, helping to maintain the health and function of the temporomandibular joints. In this report, MEAW and a reverse curve archwire were used to level and flatten the Spee curve. Consequently, the correction of the Spee curve in this case report was achieved after treatment (Fig. 6). The reconstruction of the occlusal plane is mainly achieved by using MEAW together with the vertical elastics on the anterior teeth. The horizontal loop of MEAW provides a continuous low orthodontic force. In addition, the horizontal loop separates one tooth from the other at the various contact points. In other words, we can bend the arch wire with a tip back or make a stepped bend and apply force to each tooth individually to shorten the treatment time. The occlusal plane (OP-MP) has decreased slightly by 0.7°.

The condition of the TMJ is one of the important contents that orthodontists should pay attention to throughout the orthodontic treatment process [11]. Among the anatomical control factors of mandibular movement, the guidance of the anterior incisors and the guidance of the canines play an essential role in the formation of a stable ICP and the control of mandibular extension and lateral movement [12]. Patients with anterior open-bite may experience unstable ICP and unbalanced occlusion due to long-term loss of anterior incisor guidance and lateral canine guidance, resulting in impaired masticatory muscle strength and muscle dysfunction during functional movement, as well as uncoordinated mandibular movement. The presence of traumatic oral and maxillofacial movements can lead to TMJ disorders and promote the development of TMD [11]. TMD can lead to various changes in oral function, such as tongue disorders, limited border movement, and changes in masticatory environment that exacerbate the extent of malocclusion [13]. Ultimately, a vicious cycle of mutual promotion of malocclusion and TMD is created. It is necessary to comprehensively understand the patient’s temporomandibular joint condition before alleviating the effects of occlusal factors on the temporomandibular joint in clinical practice. And then determine whether occlusal factors have a negative influence on the occurrence of TMD, make an assessment of the corresponding prognosis, and finally carry out occlusal adjustment treatment. Through orthodontic treatment, we establish a stable occlusal environment for patients, and thus promote the balance of the oral and maxillofacial system [14].

Before orthodontic treatment, the girl had a history of restricted mouth opening. We conducted a condylar trajectory analysis on this patient before and after orthodontic treatment. The results showed that the patient’s condylar border movement was still within the normal range after orthodontic treatment. By establishing posterior occlusal support and anterior guidance through orthodontic treatment, the functional movement of the condyle was improved. By comparing the bilateral TMJ films extracted from this patient’s CBCT before and after orthodontic treatment, it was found that the resorbed condyles showed some degree of remodeling [15]. Shen et al. showed that the reconstruction of the TMJ by mandibular relocation and the reconstruction of the periodontal tissues by establishing occlusion are the histological basis for stabilizing the new mandibular position [16].

The present case in this study was achieved by lowering the posterior teeth, retracting and extruding the maxillary anterior teeth and cooperating with intermaxillary traction, which ultimately result in normal occlusion coverage of the anterior teeth. At the same time, the depression of the posterior teeth led to an anticlockwise rotation of the occlusal plane and an improvement of the occlusion. During the leveling and alignment phase, the MEAW produced a intrusive force on the posterior molars and an extrusive force on the incisors [17]. Vertical elastics in the anterior region were used and were significant for improving vertical dimensional control of the anterior region. These therapeutic methods were beneficial for increasing the overbite by counterclockwise rotation of the occlusal plane [18]. According to Sato et al., the occlusal plane is steeper in individuals with Class II malocclusions and flatter in individuals with Class III malocclusions than in the Class I occlusion group [19, 20]. Therefore, the development of treatment plans should be based on etiologic considerations and control of the occlusal plane [21].

## Conclusion

Considering the maxillofacial vertical growth may associated with poor oral habits, much attention should be paid to the early intervention with myofunctional therapy. Early diagnosis and intervention in patients with poor tongue habits can be decisive. Intrusion of the maxillary incisor makes a difference during the camouflage treatment of the malocclusion. Through establishing a stable occlusal environment for patients, the balance of the oral and maxillofacial system including TMJ can be achieved.

## Data Availability

The authors confirm that the data supporting the findings of this study are available within the article.

## Abbreviations

ICR: Idiopathic condylar resorption
TMJ: Temporomandibular joint
